# Cu- and Fe-Doped Ni-Mn-Sn Shape Memory Alloys with Enhanced Mechanical and Magnetocaloric Properties

**DOI:** 10.3390/ma17133172

**Published:** 2024-06-28

**Authors:** Siyao Ma, Xuexi Zhang, Guangping Zheng, Mingfang Qian, Lin Geng

**Affiliations:** 1 School of Materials Science and Engineering, Harbin Institute of Technology, Harbin 150001, China; siyao0713.ma@connect.polyu.hk (S.M.);; 2 Department of Mechanical Engineering, The Hong Kong Polytechnic University, Hong Kong, China

**Keywords:** Ni-Mn-Sn alloys, magnetic properties, mechanical properties, magnetocaloric effect, first-principles calculation

## Abstract

Ni-Mn-Sn-based ferromagnetic shape memory alloys (FSMAs) are multifunctional materials that are promising for solid-state refrigeration applications based on the magnetocaloric effect (MCE) and elastocaloric effect (eCE). However, a combination of excellent multi-caloric properties, suitable operating temperatures, and mechanical properties cannot be well achieved in these materials, posing a challenge for their practical application. In this work, we systematically study the phase transformations and magnetic properties of Ni_50−x_Mn_38_Sn_12_Cu_x_ (x = 0, 2, 3, 4, 5, and 6) and Ni_50−y_Mn_38_Sn_12_Fe_y_ (y = 0, 1, 2, 3, 4, and 5) alloys, and the magnetic-structural phase diagrams of these alloy systems are reported. The influences of the fourth-element doping on the phase transitions and magnetic properties of the alloys are elucidated by first-principles calculations. This work demonstrates that the fourth-element doping of Ni-Mn-Sn-based FSMA is effective in developing multicaloric refrigerants for practical solid-state refrigeration.

## 1. Introduction

Ni-Mn-Sn-based ferromagnetic shape memory alloys (FSMAs) are one of the most important solid-state refrigeration materials due to their first-order martensitic and second-order magnetic transformations. Moreover, they are not toxic and possess environmental friendliness and cost-effectiveness. Nonetheless, it is crucial to address the issues of inherent brittleness and low working temperature of Ni-Mn-Sn-based FSMAs, and it is imperative that their magnetocaloric effect (MCE) and elastocaloric effect (eCE) performance be greatly improved. The introduction of Cu into Ni-Mn-based alloys promotes the coupling of martensitic and magnetic transformations, leading to a significant magnetocaloric effect and magnetically induced strain [1,2,3]. At the same time, the doping of Cu can improve the plasticity of Ni-Mn-based alloys while reducing material costs. In 2010, Wang et al. [4] obtained a fracture strain of 22% and a compressive strength of 878 MPa in Ni_50_Mn_25_Ga_17_Cu_8_ alloys. The doping of the ferromagnetic element Fe in Ni-Mn-Ga alloys may improve the magnetic properties of the alloys, which has attracted much attention [5]. Similar to Cu doping, Fe doping can also improve the ductility of the alloy [6]. However, Fe doping is more prone to forming a second phase [7]. In 2001, Cherechukin et al. [8] found that adding a small amount of Fe to polycrystalline Ni-Mn-Ga alloys improves their plasticity without compromising their magnetocaloric properties.

Recently, it has been suggested that the magnetic properties of Ni-Mn-Sn-based alloys can be improved through doping of the fourth element [9,10,11,12]. Such a doping method enables the regulation of the martensitic transformation (MT) temperature (*T_M_*) so that the conditions of occurrence of eCE, i.e., martensitic Curie temperature (*T_M_^C^*) < *T_M_* < austenite Curie temperature (*T_A_^C^*), can be fulfilled. Consequently, magneto-structural coupling can be achieved, resulting in a significant difference in magnetization (Δ*M*) during the martensitic phase transformation. The driving force behind the magnetic transition is attributed to Zeeman energy *E_zeeman_ ~*Δ*M∙H*, where *H* is the applied magnetic field, and materials with larger Δ*M* are anticipated to exhibit better magnetic-field-induced martensitic transformations (MT) and improved MCE properties [13].

Previous studies have emphasized the sensitivity of phase transition temperature and Curie temperature of Heusler alloys with shape memory effect to material compositions, including non-stoichiometric ratios and contents of doping elements [14,15,16]. Ye et al. [17] prepared Ni_2_Mn_1+x_Sn_1−x_ (x = 0, 0.12, 0.36, 0.42) polycrystalline samples and employed first-principles calculation to study the valence band structures at different contents *x*. The results demonstrated that no martensitic transformation occurred in the alloys with x ≤ 0.25, consistent with experimental findings. With increasing x, the peak of spin-down 3D-electronic density of states for the high-temperature cubic phase gradually migrated towards the Fermi level E_F_. This peak was formed by the hybridization of Ni 3D electrons with those of excess Mn atoms occupying Sn sites, creating an antiferromagnetic coupling. He et al. [18] used density functional theory calculations to study the effects of Mn and Co doping on the structure, magnetisms, and phase transitions of Ni_2_MnZ (Z = In, Sn, and Sb) alloys. The calculation of the formation energy showed that the excess Mn preferentially occupied the Z atomic site, and the energy difference between the two phases increased with increasing Mn doping. Co atoms, on the other hand, preferred to occupy the Ni atomic site, and the energy difference between the two phases decreases with increasing Co doping content, consistent with the experiment results that the martensitic transformation temperature decreased with increasing Co doping content.

To explore a novel Ni-Mn-Sn-based magnetic alloy with large magnetocaloric effects and exceptional mechanical properties, a series of Ni_50-x_Mn_38_Sn_12_Cu_x_ (x = 0, 2, 3, 4, 5, and 6) and Ni_50−y_Mn_38_Sn_12_Fe_y_ (y = 0, 1, 2, 3, 4, and 5) alloys are prepared. Subsequently, the microstructure, variations on phase transition parameters, mechanical properties, and magnetic-structural phase diagrams of the Ni_50−x_Mn_38_Sn_12_Cu_x_ and Ni_50−y_Mn_38_Sn_12_Fe_y_ alloys are studied. The influences of the fourth-element doping on the phase transitions and magnetic properties of the alloys are elucidated by first-principles calculations. The results demonstrate that Ni-Mn-Sn-based alloys doped with Cu or Fe can be excellent working materials for room-temperature magnetocaloric refrigeration.

## 2. Experimental and Computational Methods

A series of polycrystalline parent ingots of Ni_50−x_Mn_38_Sn_12_Cu_x_ (x = 0, 2, 3, 4, 5, and 6) and Ni_50−y_Mn_38_Sn_12_Fe_y_ (y = 0, 1, 2, 3, 4, and 5) alloys were prepared by vacuum induction melting of high-purity raw materials in argon atmosphere inside a water-cooled copper mold. Considering the high volatility of Mn in comparison with other constituent alloy elements, an extra 2 wt.% of the total mass of Mn was added to make up for its loss during melting. The cylindrical ingot of 9 mm in diameter and 40 mm in height was placed within an evacuated quartz tube. Then it was annealed at 1173 K for 24 h before being quenched in water.

The MT temperatures were analyzed by differential scanning calorimetry (DSC, Q200, TA Instruments Inc., New Castle, DE, USA) with a heating and cooling rate of 10 K/min. The starting and finishing temperatures of the martensitic and its reverse transformation were determined by the double tangent method. The crystal structure was identified by X-ray diffraction (XRD, D500 S equipment, Bruker, Billerica, MA, USA) with Cu Kα radiation (λ = 1.5406 Å), which was operated at 45 kV and 250 mA. The microstructure was analyzed by an optical microscope (OM, PMG3, Olympus Corporation, Tokyo, Japan) under polarization mode and a scanning electron microscope (SEM, SUPRA 55 SAPPHIRE, ZEISS, Oberkochen, Deutschland). The magnetization-temperature (*M-T*) tests were performed on a superconducting quantum interference device (SQUID, MPMS, Quantum Design, San Diego, CA, USA). The *M-T* plots were recorded under magnetic fields of 0.02 and 5.0 T, respectively, at a heating and cooling rate of 5 K/min. The loop process method was used to estimate the isothermal magnetization curves in order to rule out the influences of the residual phase-dependent thermal and magnetic histories. The M-H curves were measured at different test temperatures (T_test_) under an external magnetic field up to 5.0 T. The sample was initially rapidly cooled to 150 K in zero field to achieve a full weak magnetic martensitic state, then heated to (T_test_−10) K at 10 K/min, and finally heated to T_test_ at 1 K/min, and kept at T_test_ for 5 min before the M-H measurement. Mechanical properties were performed on a mechanical testing machine (5966, Instron Corporation, Canton, OH, USA) using samples with dimensions of Φ3 × 4.5 mm^3^ as cut from the ingots. The loading speed of the compressive test is 3.3 × 10^−4^·s^−1^.

The effects of doping of transition metal Cu or Fe on the crystal structure, energy, and density of states of Ni-Mn-Sn alloys were systematically studied by first-principles calculation using the Vienna Ab-initio Software Package (VASP, version 5.4.4) [19] package. The exchange correlation was based on Perdew-Burke-Enzerh (PBE) functional in the generalized gradient approximation (GGA) [20] framework, with spin-polarized treatment for electrons. Using the plane wave pseudopotentials, the interaction between ions and electrons was described by the projected augmented wave (PAW) method [21]. The electronic configurations of the involved elements were Ni(3d84s2), Mn(3d64s1), Sn(5s25p2), Cu(3d104s1), Fe(3d64s2). The convergence tolerance for the calculations was selected as 1 × 10^−5^ eV/atom, and the cutoff of the energy plane wave was set to be 600 eV. The Brillouin zone was sampled using the Monkhorst-Pack method, and there is a 15 × 15 × 15 k-point mesh for a unit cell containing 16 atoms with the L2_1_ structure.

## 3. Results and Discussion

### 3.1. Microstructure

**Figure 1:**
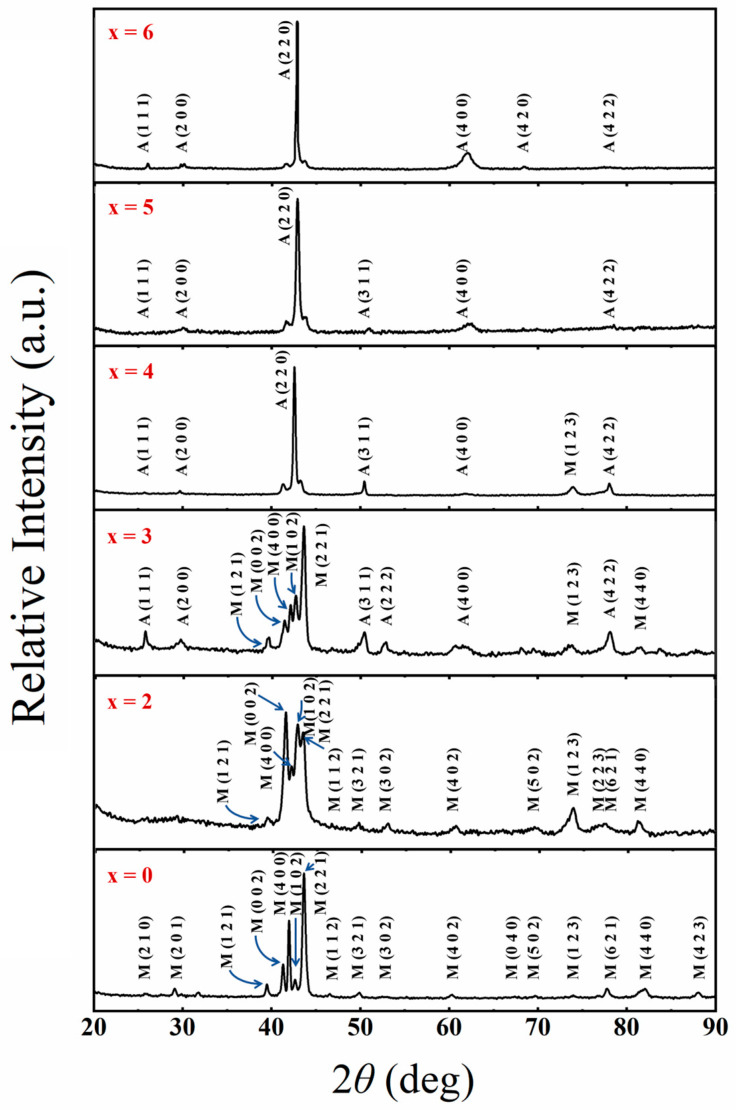
XRD patterns and miller indices of Ni_50−x_Mn_38_Sn_12_Cu_x_ (x = 0, 2, 3, 4, 5, and 6) alloys measured at room temperature. (M and A represent the martensitic and austenitic phases, respectively.)

Figure 1 shows the X-ray diffraction patterns of Ni_50−x_Mn_38_Sn_12_Cu_x_ (x = 0, 2, 3, 4, 5, and 6) measured at room temperature, suggesting that the structures of these alloys change with increasing concentrations of Cu. The Ni_50−x_Mn_38_Sn_12_Cu_x_ (x = 0, 2) alloy matrix at room temperature is a martensite structure. Compared with the XRD patterns of Ni-Mn-Sn alloys reported by Krenke et al. [22], the characteristic diffraction peaks can be identified as 4O-modulated martensitic structures. In the Ni_47_Mn_38_Sn_12_Cu_3_ alloy, the superlattice diffraction peak A(311) appears, and the alloy matrix is a mixed phase of L2_1_ austenite and martensite at room temperature. When the Cu content is 4 at.%, the characteristic peak A(220) of austenite appears. In the Ni_50−x_Mn_38_Sn_12_Cu_x_ (x = 5, 6) alloys, the diffraction peak of the martensite phase is completely absent, indicating a complete transformation of martensite into austenite through a reverse martensite phase transformation at room temperature.

**Figure 2:**
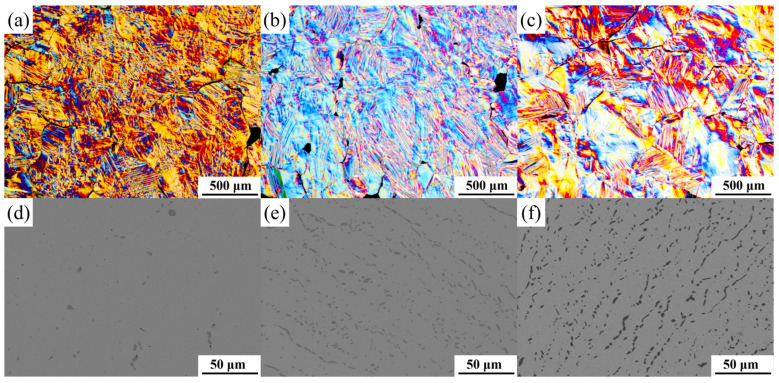
Polarized optical images in Ni_50−y_Mn_38_Sn_12_Fe_y_ (y = 0, 1, and 2) alloys and back scattered electron (BSE) micrographs in Ni_50−y_Mn_38_Sn_12_Fe_y_ (y = 3, 4, and 5) alloys. (**a**) y = 0; (**b**) y = 1; (**c**) y = 2; (**d**) y = 3; (**e**) y = 4; (**f**) y = 5.

Figure 2a displays a polarized metallographic image of an undoped ternary alloy, Ni_50_Mn_38_Sn_12_, revealing the presence of martensitic variants of various scales. Under polarized light, the different colors indicating the martensite variants of the Ni-Mn-Sn alloy suggest distinct crystallographic orientations among the variants. The lamellar and spearhead-shaped martensite structures exhibit a self-cooperative arrangement, with visible fine substructures inside. Furthermore, the grains appear to be relatively coarsened after undergoing heat treatment, with the average grain size of the central equiaxed crystal measured at about 270 μm.

The microstructures of the Ni_50−y_Mn_38_Sn_12_Fe_y_ alloys with y = 0, y = 1, and y = 2 at room temperature suggest that they are single-phase solid solutions. However, as the Fe doping content increases, the microstructure gradually transforms into a two-phase mixed microstructure of austenite and martensite. The martensite phase extends from one side of the austenite grain boundary towards the interior of the austenite grain, forming a distinct spearhead-shaped deformed morphology, which is attributed to the process of martensite formation. Initially, self-cooperative variants nucleate and grow at the grain boundaries, stimulating the growth of other variants. As the phase transition progresses, spearhead-like self-cooperative morphologies develop at the ends of the variants.

Figure 2d–f shows the backscattered electron images of the Ni_50−y_Mn_38_Sn_12_Fe_y_ alloys after subjected to heat treatment. It can be observed that when y = 3, a small amount of γ phase precipitates appear locally. As for the alloy with y = 4, the γ phase is noticeably larger at the grain boundaries compared with itself within the grain, indicating that the γ phase nucleates and grows primarily at the grain boundaries. Fe doping plays a significant role in refining the grain sizes, with the average grain size of the central equiaxed crystals measured to be approximately 40 μm. As the Fe content increases, the quantity and grain size of the second phase gradually increase. Furthermore, the distribution of the second phase expands from the grain boundaries into the grain interiors. Although the second phase does not directly participate in the martensitic transformation, it greatly improves the alloy’s plasticity. Therefore, a suitable amount of second-phase precipitation can influence the matrix composition, regulate the magneto-structural phase transition of the alloys, and enhance their mechanical properties.

### 3.2. Optimization of Phase-Transition Parameters

In the compositional design of Ni-Mn-Sn-based alloys, the main objective is to achieve a phase transition near room temperature of the alloys. By replacing the Ni element in the Ni_50_Mn_38_Sn_12_ FSMA with Cu or Fe, the phase transition parameters can be adjusted to optimize its MCE and eCE performance.

**Figure 3:**
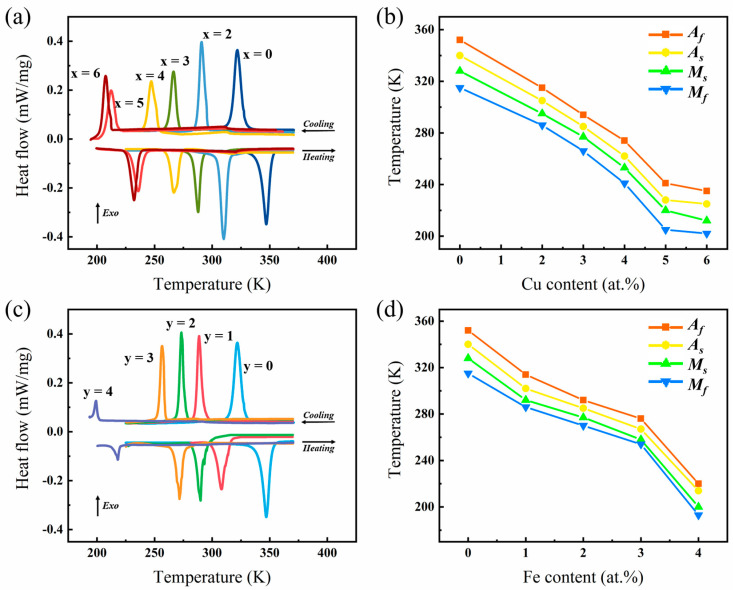
Martensite transformation characteristic temperatures of Ni_50−x_Mn_38_Sn_12_Cu_x_ (x = 0, 2, 3, 4, 5, and 6) and Ni_50−y_Mn_38_Sn_12_Fe_y_ (x = 0, 1, 2, 3, 4, and 5) alloys. (**a**,**c**) DSC curves; (**b**,**d**) Phase transition characteristic temperatures versus doping element content.

The DSC curves and the characteristic phase transition temperatures extracted from the DSC results are shown in Figure 3. The doping of Cu or Fe can shift the phase transition characteristic temperature of the Ni-Mn-Sn-based alloy to a relatively lower temperature. When x reaches 3 or y reaches 2, the phase transition temperature of the alloy drops slightly below room temperature. It is thus suggested that doping Fe or Cu in Ni-Mn-Sn-based alloys can achieve the design goal of adjusting MT.

**Figure 4:**
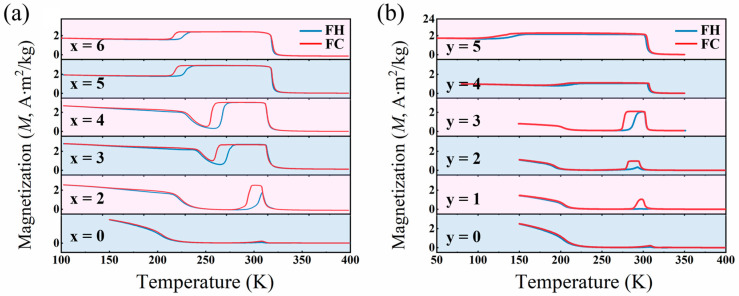
Temperature-dependent magnetization for (**a**) Ni_50−x_Mn_38_Sn_12_Cu_x_ alloys and (**b**) Ni_50−y_Mn_38_Sn_12_Fe_y_ alloys.

Figure 4 shows the temperature-dependent magnetization (M~T) curves for Ni_50−x_Mn_38_Sn_12_Cu_x_ (x = 0, 2, 3, 4, 5, and 6) and Ni_50−y_Mn_38_Sn_12_Fe_y_ (y = 0, 1, 2, 3, 4, and 5) alloys measured at a magnetic field of 0.05 T. The M~T curves were performed in the temperature range of 50–400 K in field-cooled cooling (FC) and field-cooled heating (FH) sequences, respectively. Comparing the values of characteristic phase transition temperatures measured by DSC with those determined from the thermomagnetic curves as measured under an external field of 0.05 T, it can be found that the measurement results of the two methods are basically consistent. It can be clearly seen from the figures that the magnetism of the Ni_50_Mn_38_Sn_12_ alloy is very weak near the phase transition temperature. With Cu or Fe doping, the magnetism of the alloy tends to increase in the phase transition region, indicating that the Cu or Fe-doped Ni_50_Mn_38_Sn_12_ alloys can enhance the magnetization of the alloys.

As shown in Figure 4a, the Ni_48_Mn_38_Sn_12_Cu_2_ alloy undergoes an incomplete austenite transformation with a jump in magnetization. When the Cu doping reaches 3 at.%, the alloy undergoes a complete austenite transformation. As shown at the FC curve of the Ni_47_Mn_38_Sn_12_Cu_3_ alloy with an initial high-temperature paramagnetic austenite phase, when the temperature drops to 315 K (the Curie temperature of the austenite phase, *T_C_^A^*), the magnetization first shows a rapid increase, and the alloy enters the ferromagnetic austenite state. With a further decrease in temperature, the magnetization decreases sharply at 262 K (the martensitic transformation initiation temperature, *Ms*) until the magnetization reaches a minimum value, indicating the completion of the martensitic transformation. In the lower temperature region, the magnetization gradually increases with decreasing temperature since the martensite transforms from a paramagnetic state to a ferromagnetic state at 240 K (the Curie temperature of the martensitic phase, *T_C_^M^*). When the doping amount of Cu increases to 5 at.% and above, it can be seen from the FC curve that the process of transformation of paramagnetic austenite into ferromagnetic austenite is the same as that of other components around the Curie temperature. With the decrease in temperature, the martensitic transformation directly results in ferromagnetic martensite. Notwithstanding, there still exists a noticeable difference in saturation magnetization (Δ*M*) between austenite and martensite driven by a magnetic field, due to the distinct ferromagnetic states of these phases.

It is important to highlight that in the magnetization curve of the Ni_49_Mn_38_Sn_12_Fe_1_ alloy, distinct behaviors are observed. As shown in the FC curve, the transformation from paramagnetic austenite to ferromagnetic austenite occurs in the high-temperature region, followed by the transformation from ferromagnetic austenite to paramagnetic martensite. On the other hand, at the FH curve, a “hump” shape appears during the reverse martensite transformation. According to the DSC results, it can be inferred that the magnetic transformation blocks the reverse martensite transformation, resulting in the “hump” shape, at the FH curve. Partial coupling between the martensitic phase transition and the magnetic transition enables MCE with continuous transformations.

### 3.3. The Improved Magnetic Properties

**Figure 5:**
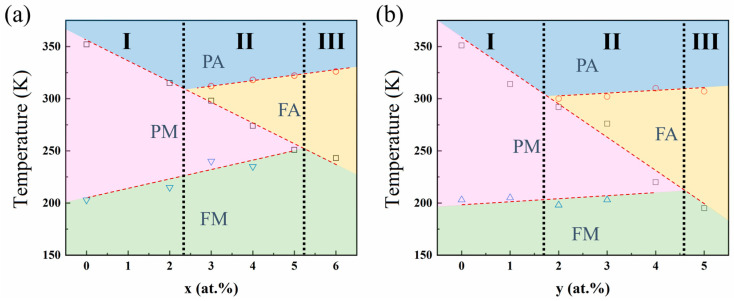
Structural-magnetic phase diagram of (**a**) Ni_50−x_Mn_38_Sn_12_Cu_x_ (x = 0, 2, 3, 4, 5, and 6) and (**b**) Ni_50−y_Mn_38_Sn_12_Fe_y_ (y = 0, 1, 2, 3, 4, and 5) alloys. (Triangle represents the martensite Curie temperature determined from the M-T curve, square and circle represent the martensite transformation temperature and austenite Curie temperature determined from the DSC curve, respectively.)

Based on the phase transition temperature, martensitic Curie temperature, and austenite Curie temperature that have been determined from DSC curves and M-T curves, the magnetic-structural phase diagram of Ni_50−x_Mn_38_Sn_12_Cu_x_ (x = 0, 2, 3, 4, 5, and 6) and Ni_50−y_Mn_38_Sn_12_Fe_y_ (y = 0, 1, 2, 3, 4, and 5) alloys is divided into four regions. They are paramagnetic austenite, ferromagnetic austenite, paramagnetic martensite, and ferromagnetic martensite, respectively. Additionally, the phase diagram can be divided into three regions according to the changes in magnetic properties during the phase transition, as shown in Figure 5.

There are three regions in the diagram, described as follows: Region I: The transformations of PA→PM→FM occur with decreasing temperature, and the martensitic phase transformation from paramagnetic austenite to paramagnetic martensite occurs; there is no magnetic transformation. Region II: With decreasing temperature, the transformations of PA→FA→PM→FM occur, and the martensitic phase transformation from ferromagnetic austenite to paramagnetic martensite occurs. Since there is a magnetic transformation in this region, the magneto-structural coupling could occur, which has been investigated in previous work on Ni-Mn-X (X = In, Sn, Sb, and Al) ferromagnetic alloys [23,24,25]. The coupling is found to be responsible for the excellent magnetic actuation performance observed in the alloys, and the doped Ni-Mn-Sn alloys are expected to exhibit improved magnetic properties. Region III: the transformations of PA→FA→FM occur with a decrease in temperature, and the martensitic transformation occurs in the ferromagnetic martensite. Since the ferromagnetic states of austenite and martensite are different, a noticeable Δ*M* between austenite and martensite under an applied magnetic field could occur.

**Figure 6:**
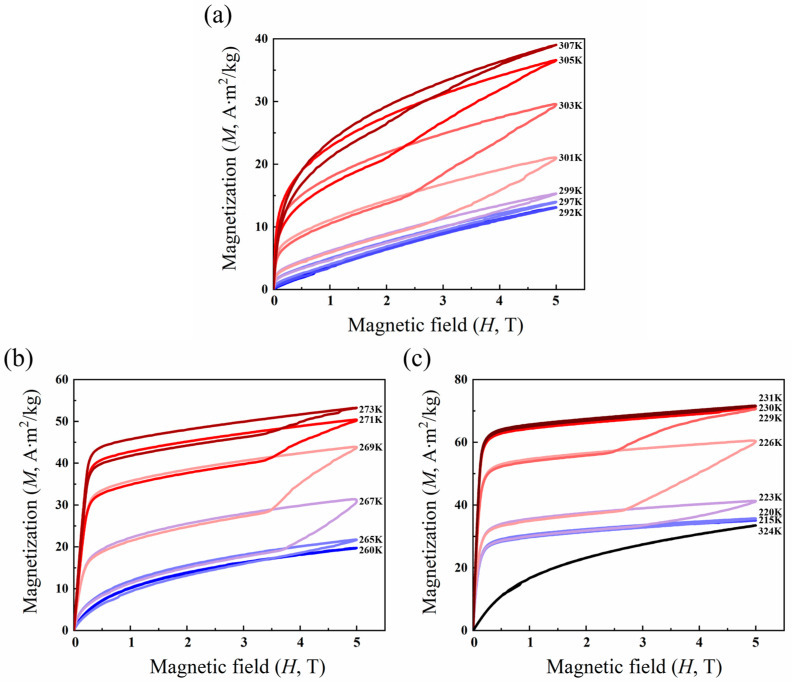
Selected M-H curves under 5.0 T at different temperatures for Ni_50−x_Mn_38_Sn_12_Cu_x_ alloy (**a**) x = 2; (**b**) x = 3; and (**c**) x = 5.

According to Figure 6a, it can be seen that the alloy sample with x = 2 exhibits characteristics of paramagnetic magnetization in the high-temperature martensite. The magnetization curve presents an obvious ring-shaped characteristic at 301 K, and the magnetization of the alloy sample gradually increases to ~10 emu/g when the applied magnetic field gradually increases to ~2.8 T, which corresponds to the magnetization of martensite in the alloy. As the applied field is further increased to as high as 5 T, it is difficult to reach the magnetization saturation for the alloy, which is attributed to the existence of excess Mn atoms in the Mn-rich Ni-Mn-Sn-based alloy. In comparison with the Ni-Mn-Sn alloy with a stoichiometric ratio, the excess Mn atoms have a tendency to occupy the Sn atomic sites, leading to the formation of antiferromagnetic coupling between those in-situ Mn atoms and the Mn atoms occupying the Sn sites.

Figure 6b illustrates the magnetization curves of the alloy samples with x = 3 at different temperatures. It is observed that the alloy is more easily magnetized to saturation as compared with the alloy with x = 2. Although the doping of Cu atoms can alternate the antiferromagnetic Mn-Mn interaction into the ferromagnetic one, the Cu content in this alloy sample is not sufficient to complete such a transformation and only partially weakens the Mn-Mn interaction. As a result, the alloy exhibits a short-range ferromagnetic order.

In the alloy with x = 5, the hysteresis loop disappears when the temperature is increased to 230 K, and the alloy is in the ferromagnetic austenite parent phase. At a lower magnetic field, the magnetization increases sharply and then reaches saturation. When the temperature is 324 K, close to the austenite Curie temperature of the alloy, the magnetic properties will gradually change from ferromagnetic to paramagnetic.

**Figure 7:**
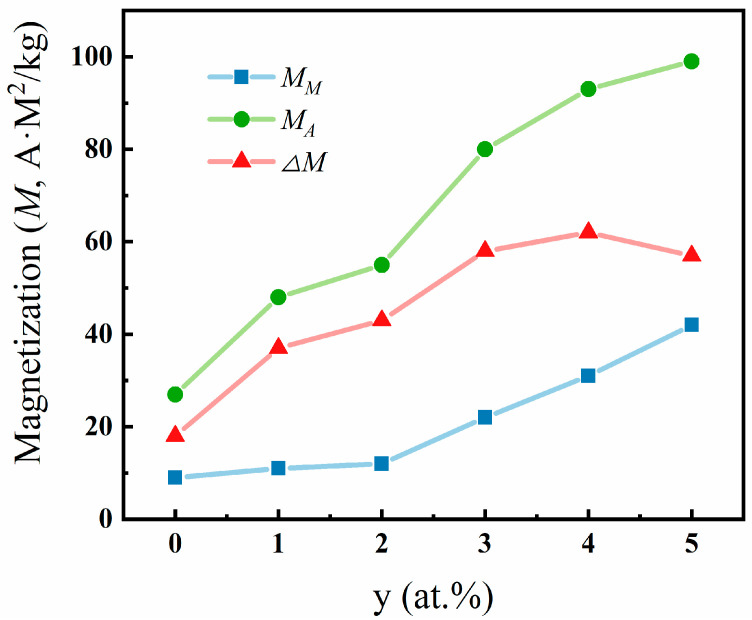
The magnetization of full austenite (*M_A_*) and full martensite (*M_M_*) phases and the magnetization difference related to martensitic transformation Δ*M* = *M_A_* − *M_M_* as a function of Fe content under 5 T in Ni_50−y_Mn_38_Sn_12_Fe_y_ (y = 0, 1, 2, 3, 4, and 5) alloys.

Under the application of an external magnetic field of 5.0 T, the variations of magnetization of austenite (*M_A_*), magnetization of martensite (*M_M_*), and Δ*M = M_A_ − M_M_* with Fe content are shown in Figure 7. It should be emphasized that *M_M_* increases significantly with increasing Fe content when y > 3, while *M_A_* increases more rapidly, resulting in an increased magnetization difference Δ*M* for the two phases with increasing Fe content, which becomes saturation at y = 4. It is thus confirmed that the doping of Fe enhances the magnetic properties of austenite and increases the saturation magnetization difference between the austenite and martensite phases, and the magnetic field-driven martensitic transformation is more obvious.

### 3.4. The Improved Mechanical Properties

For the practical application of Ni-Mn-Sn alloys in solid-state refrigeration, it is crucial that the alloys have stable and excellent mechanical properties. To assess the impact of Cu and Fe doping on the mechanical properties of Ni_50−x_Mn_38_Sn_12_Cu_x_ (x = 0, 2, 3, 4, 5, and 6) and Ni_50−y_Mn_38_Sn_12_Fe_y_ (y = 0, 1, 2, 3, 4, and 5) alloys, compression tests on the alloys are carried out at room temperature, which are loaded to fracture.

**Figure 8:**
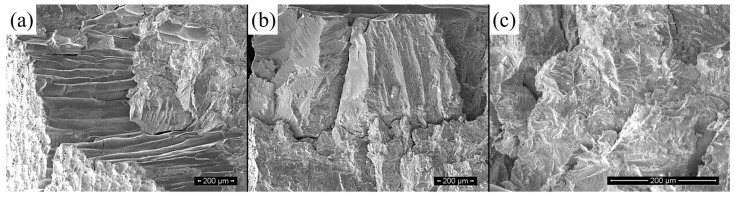
SEM images of fracture surface morphologies of Ni_50−x_Mn_38_Sn_12_Cu_x_ alloy (**a**) x = 0; (**b**) x = 2; and (**c**) x = 6.

Figure 8a shows the fracture surface morphology of the Ni_50_Mn_38_Sn_12_ ternary alloy at room temperature. It can be clearly observed that most of the fracture areas are smooth and the grain boundary interfaces are clean, suggesting a typical fracture along the grain boundary in the alloy. The SEM image shows that the alloy has poor ductility and toughness. After doping 2 at.% Cu in the alloy, the fracture edge of the Ni_48_Mn_38_Sn_12_Cu_2_ alloy can be observed, which indicates that the alloy has a certain plastic deformation before fracture. The fracture surface morphology presents a mixture of intergranular fracture and cleavage fracture, as shown in Figure 8b.

The fracture surface morphology of Ni_48_Mn_38_Sn_12_Cu_2_ is indicated in Figure 8c. Compared with Figure 8a, it can be seen that the microstructure of the Ni_48_Mn_38_Sn_12_Cu_2_ alloy is refined, the fracture mode of the alloy is mainly cleavage fracture, and only a few typical features of the morphology of intergranular fracture can be observed. A large number of ductile tearing edges appear on the fracture surface of the alloy, and the tearing edges are observed at the grain boundaries of the alloy. The fracture surface morphology of the Ni_44_Mn_38_Sn_12_Cu_6_ alloy shows river patterns and tongue-like patterns, and a small amount of dimples can be observed. It is thus suggested that there is a large plastic deformation before the fracture occurs in the alloy, and the toughening effect of the doping of Cu with a content of 6 at.% is remarkable. In addition, a further refined fracture structure can be observed, resulting in limited crack generation during the compressive fracture. It is found that even when initial cracks are generated, the subsequent growth and extension of cracks are difficult, and the fracture mode of the alloy changes to a transgranular fracture. As a result, the plasticity of the alloy is significantly improved as compared with that of the Ni_50−x_Mn_38_Sn_12_Cu_x_ alloys with x < 6 at.%.

**Figure 9:**
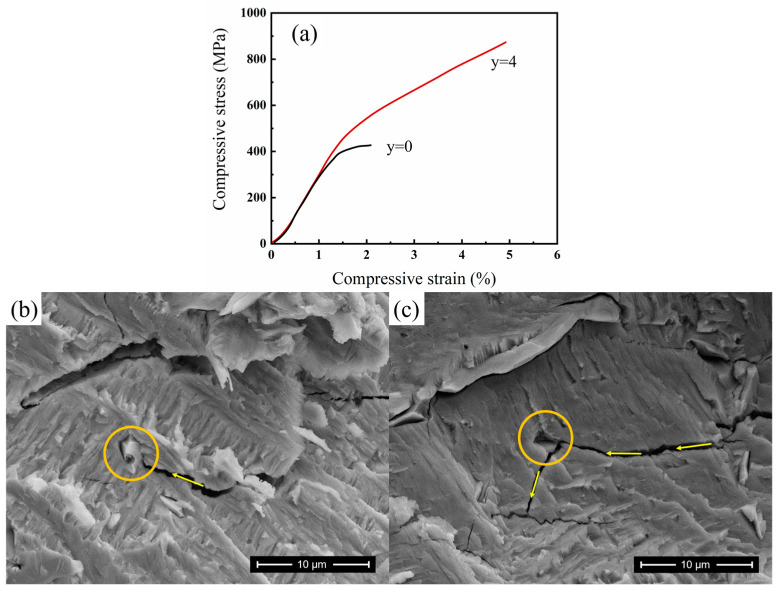
Mechanical properties and fracture surface characteristics. (**a**) Compressive stress-strain curves of Ni_50_Mn_38_Sn_12_ and Ni_46_Mn_38_Sn_12_Fe_4_. (**b**,**c**) are high-magnification optical images on surfaces of the Ni_46_Mn_38_Sn_12_Fe_4_ alloy. (The yellow circle indicates the location of a γ phase precipitate. The arrows show the direction of crack propagation.)

The mechanical properties of ternary Ni_50_Mn_38_Sn_12_ alloy samples are poor, as indicated by the stress-strain curves shown in Figure 9a. The fracture stress is about 475 MPa, and the fracture strain is only about 3.1% for the ternary alloy. The addition of Fe significantly improves the compressive ductility of Ni_46_Mn_38_Sn_12_Fe_4_, the compressive strength reaches 903 MPa, and the fracture strain reaches 5.1%. The crack propagation processes on the fracture surfaces of Ni_46_Mn_38_Sn_12_Fe_4_ alloys are shown in Figure 9b,c. Previous work shows that the Ni-Mn-Sn alloys exhibit typical cleavage-brittle fracture characteristics. In contrast, as shown in Figure 9b, it can be seen that the Ni_46_Mn_38_Sn_12_Fe_4_ alloy has a γ phase precipitate (at the position indicated by the yellow circle in the figure), which will produce plastic deformation under stress. Close to the precipitate, the crack blunts, and the stress concentration at the crack tip is released. The presence of the γ phase obstructs the crack growth process, effectively arresting its propagation.

It should be pointed out that under the aforementioned mechanism, the toughening effect can only be achieved when the γ phase precipitates are diffusely distributed; otherwise, it cannot be guaranteed that the crack initiation site contains the γ phase. As shown in Figure 9c, the γ phase precipitate indicated in the yellow circle becomes detached, and the cracks will propagate along the interface between the γ phase and the matrix, forming a tortuous fracture path, dissipating more energy, and improving the toughness of the Ni_46_Mn_38_Sn_12_Fe_4_ alloy [26].

For the Ni_46_Mn_38_Sn_12_Fe_4_ alloy, the existence of a large number of γ phase precipitates could result in a significant increase in thermal hysteresis. Such a second phase acts as a resistance to martensitic transformation, which is unfavorable to the MCE and eCE of the alloy. Therefore, the role of the second phase in the Ni-Mn-Sn-based alloy needs to be further explored, considering both its positive impact on mechanical properties and potential drawbacks in terms of multi-caloric properties.

### 3.5. First-Principles Calculation on Phase Transitions and Magnetic Properties

It is a challenging task to experimentally determine the sub-lattice position of the added fourth elements, Cu or Fe, in the doped Ni-Mn-Sn alloys. To determine the preferred occupation of the doped elements and establish accurate structural models for quaternary Ni-Mn-Sn-Cu and Ni-Mn-Sn-Fe alloys, the preferential occupation of Cu and Fe is studied from the perspective of formation energy. This approach helps to determine the most favorable positions for the doped elements in the Ni-Mn-Sn matrix.

The parent Ni_8_Mn_4_Sn_4_ alloy possesses a face-centered cubic structure with a space group of $Fm\bar{3}m$, but this particular alloy does not undergo the austenite-to-martensite transformation. Previous studies have indicated that the martensitic transformation can only be observed in off-stoichiometric Ni_8_Mn_4+x_Sn_4−x_ (x ≥ 2) Mn-rich alloys [27,28]. For fist-principles calculation, the cubic L2_1_ structure is established for Ni_7_Cu_1_Mn_6_Sn_2_, Ni_6_Cu_2_Mn_6_Sn_2_, and Ni_7_Fe_1_Mn_6_Sn_2_ based on the crystal structure of the Ni_8_Mn_6_Sn_2_ alloy, as shown in Figure S1 (see Supplementary Information), and the unit cell comprises 16 atoms. Three possible atomic substitutions are considered: (1) direct substitution of Ni atoms by doping atoms. (2) Doping atoms occupying the positions of Mn atoms, while Mn atoms further replace Ni atoms. (3) Doping atoms occupying the Sn atoms, which in turn replace Ni atoms.

To determine the ground state of the austenite phase in the Ni-Mn-Sn alloys doped with the fourth element and investigate its relevant crystal structure-related properties, the effect of Cu and Fe doping on the austenite phase is examined. The total energy of the austenite phase in the Ni_8_Mn_6_Sn_2_, Ni_7_Cu_1_Mn_6_Sn_2_, Ni_6_Cu_2_Mn_6_Sn_2_, and Ni_7_Fe_1_Mn_6_Sn_2_ alloys is calculated, and its relationship with the lattice constant is determined. In off-stoichiometric Ni_8_Mn_4+x_Sn_4−x_ (x ≥ 2) Mn-rich alloys, excessive Mn atoms occupying the Sn atomic positions exhibit either parallel or antiparallel alignments with the original magnetic moments of Mn atoms, which are referred to as ferromagnetic (FM) and antiferromagnetic (AFM) magnetic configurations, respectively. Therefore, two different magnetic configurations, namely ferromagnetism and antiferromagnetism, are taken into account in the calculations.

**Figure 10:**
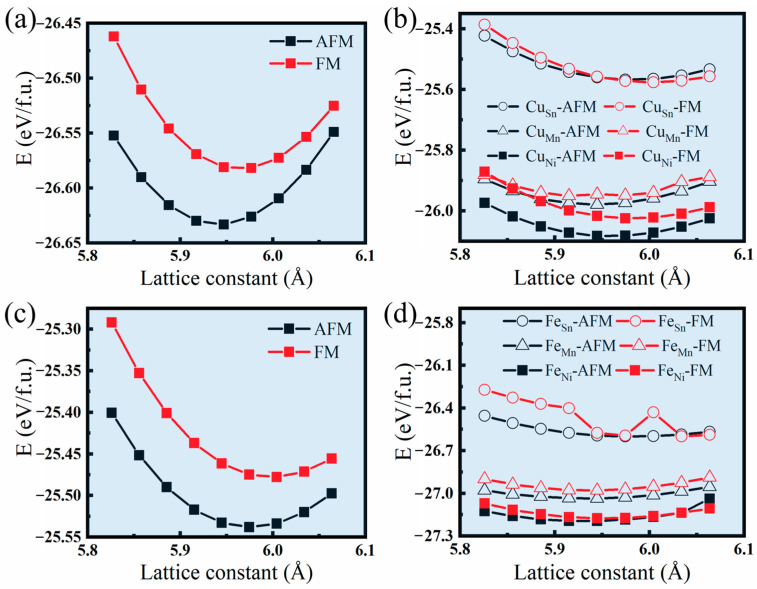
The total energies as functions of the lattice constant for FM and AFM configurations of (**a**) Ni_8_Mn_6_Sn_2_, (**b**) Ni_7_Cu_1_Mn_6_Sn_2_, (**c**) Ni_6_Cu_2_Mn_6_Sn_2_, (**d**) Ni_7_Fe_1_Mn_6_Sn_2_ in the austenitic phase.

Figure 10 shows the total energy of the undoped alloy Ni_8_Mn_6_Sn_2_, the Cu-doped alloys Ni_7_Cu_1_Mn_6_Sn_2_, Ni_6_Cu_2_Mn_6_Sn_2_, and the Fe-doped alloy Ni_7_Fe_1_Mn_6_Sn_2_ as a function of lattice constant and magnetic configurations. It can be seen from Figure 10a that the minimum value of the total energy of the antiferromagnetic state is significantly lower than that of the ferromagnetic state, and the equilibrium lattice constant of the alloy is 0.593 nm. Therefore, the ground state of the Ni_8_Mn_6_Sn_2_ parent phase is determined to be an antiferromagnetic state.

Figure 10b shows that the total energy-lattice constant curves for the ferromagnetic and antiferromagnetic states shift upward simultaneously after 6.25 at.% Cu is substituted for Ni in the alloy. Still, the lowest total energy of the alloy in the antiferromagnetic state is lower than that in the ferromagnetic state, and the equilibrium lattice constant of the alloy in the ground state is slightly increased to 0.595 nm. It can be seen that the total energy of the alloy with Cu substituting Sn and then Ni is higher than that with Cu substituting Mn and then Ni. The total energy of the alloy with Cu directly substituting Ni is the lowest, and the lower formation energy means that the configuration is more thermodynamically stable. These findings confirm that when 6.25 at.% of Cu is doped into the alloy system, the Cu atoms tend to directly occupy the sublattice sites previously occupied by Ni atoms. Based on the fact that martensitic transformation is a non-diffusion process, the neighboring relationships between atoms before and after the transformation are determined once the occupancy modes of the fourth element in the doped austenite phase are established and the occupancy information of the austenite phase can be obtained. Subsequently, other properties of the alloy can be further calculated. Figure 10c shows the total energy-lattice constant curves for the ferromagnetic and antiferromagnetic states after 12.5 at.% Ni is replaced by Cu in the alloy, which continue to progress upward with increasing Cu doping in the alloy. The lowest total energy of the alloy in the antiferromagnetic state is lower than that in the ferromagnetic state, and the equilibrium lattice constant of the alloy in the ground state continues to increase to 0.597 nm.

Figure 10d shows the simultaneous downward shift of the total energy-lattice constant curve for the ferromagnetic and antiferromagnetic states after 6.25 at.% Fe replaces Ni in the alloy. The lowest total energy of the alloy in the antiferromagnetic state is lower than that in the ferromagnetic state, and the equilibrium lattice constant of the alloy in the ground state is still 0.595 nm. It can be seen that the total energy of the alloy with Fe replacing Sn and then replacing Ni is higher than that with Fe replacing Mn and then replacing Ni, while the total energy of the alloy with Fe directly replacing Ni is the lowest. Therefore, it can be determined that in the alloy there is a direct substitution of Fe for Ni. When the lattice constant exceeds 0.60 nm, the curves for the antiferromagnetic and ferromagnetic states cross with each other, indicating that Fe doping promotes the stability of the ferromagnetic state. Moreover, the effect of Fe doping is significantly more pronounced than that of Cu doping at the same doping content.

The aforementioned results indicate that the addition of Cu or Fe is beneficial for the magnetic properties of the austenite phase. Both elements enhance the stability of the ferromagnetic state in the parent phase to a certain degree. Doping an appropriate amount of Cu or Fe is suggested to be an effective approach to controlling the magnetic exchange interactions in the Ni-Mn-Sn alloy’s parent phase. Additionally, the introduction of Fe into the alloy can lead to a shift in the ground state of the parent phase, which transforms from an antiferromagnetic state to a ferromagnetic state. Furthermore, the impact of doping on the lattice constant is evident from the curves shown in Figure 10. With increasing Cu doping, the lattice constant increases, which could be attributed to the fact that Cu possesses a full 3D orbital and a larger atomic radius in comparison with Ni, resulting in the increased spatial occupancy. On the contrary, Fe doping leads to a decrease in the lattice constant, mainly due to the much larger magnetic moment associated with Fe in comparison with Ni.

In order to study the effect of the fourth element, Cu or Fe, on the Curie temperature of Ni-Mn-Sn alloys, the total energies of paramagnetic austenite and ferromagnetic austenite in the Ni_8_Mn_6_Sn_2_, Ni_7_Cu_1_Mn_6_Sn_2_, Ni_6_Cu_2_Mn_6_Sn_2_, and Ni_7_Fe_1_Mn_6_Sn_2_ alloys are calculated, respectively. The results reveal that the total energy of the paramagnetic austenite phase is much higher than that of the ferromagnetic austenite phase. The Curie temperature (*T_C_^A^*) of the austenite phase represents the transition point between these two phases. According to Stoner’s theory, *T_C_^A^* of the austenite phase exhibits a linear relationship with the paramagnetic and ferromagnetic states’ total energy difference (Δ*E*) [29,30].

**Figure 11:**
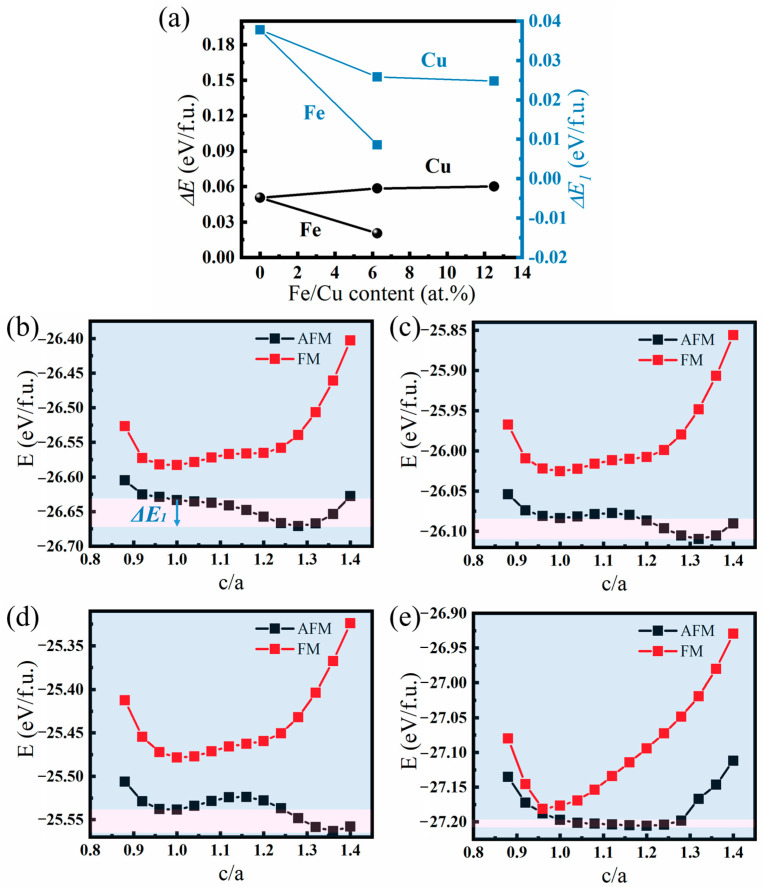
(**a**) The influence of Fe/Cu doping content on the paramagnetic and ferromagnetic austenite phases’ total energy difference (Δ*E*) and the L2_1_ and tetragonal martensitic structure energy difference (Δ*E_1_*) on the formula unit of Ni_8−x_Cu_x_Mn_6_Sn_2_ and Ni_8−y_Fe_y_Mn_6_Sn_2_ alloys. (**b**–**e**) Variation in the total energy on the formula unit as a function of c/a for Ni_8_Mn_6_Sn_2_, Ni_7_Cu_1_Mn_6_Sn_2_, Ni_6_Cu_2_Mn_6_Sn_2_, and Ni_7_Fe_1_Mn_6_Sn_2_ alloys in FM and AFM configurations, respectively.

Figure 11a illustrates the relationship between the total energy difference (Δ*E*) and composition of the paramagnetic austenite or ferromagnetic austenite phases. The plot demonstrates that as the doping content of Cu increases, Δ*E* between the paramagnetic and ferromagnetic austenite phases increases linearly. In contrast, as the doping content of Fe increases, Δ*E* decreases. Consequently, it can be inferred that the Curie temperature of the alloy increases with increasing Cu content, while it declines with increasing Fe content. The effect of adjusting *T_C_^A^* of Ni-Mn-Sn-based FSMA exhibits opposite trends for Cu and Fe doping.

The alloy’s phase stability directly affects the tendency of phase transformations and can directly affect the phase transformation temperature of the alloy. To illustrate the effect of doping elements Cu or Fe on the martensitic transformation temperature, the difference in total energy between austenite and martensite is calculated: Δ*E_1_ = E_AP(Cub.)_ − E_P(Tetra.)_*. This energy difference Δ*E_1_* between the Ni-Mn-Sn alloy’s L2_1_ austenite and the tetragonal martensite phase provides an intuitive prediction of the martensitic transformation temperature [31,32]. Figure 11a shows the relationship between the total energy difference (Δ*E_1_*) between the L2_1_ austenite and the tetragonal martensite phase and the composition, respectively. It is shown that Δ*E_1_* decreases significantly with increasing Cu or Fe content. The change in the martensite phase transformation temperature (*M_s_*) is closely linked to Δ*E_1_*. A larger value of Δ*E_1_* signifies a greater energy difference between the austenite and martensite phases, along with a higher driving force for phase transformation. Consequently, a higher value of Δ*E_1_* indicates a greater tendency for austenite to transform into martensite, as reflected in a higher martensitic transformation temperature for the alloy. At the same time, this phenomenon can be essentially explained by the densities of states, as shown in Figure S2 (see Supplementary Information).

Hence, it can be concluded that the martensitic transformation temperature of the Ni_7_Fe_1_Mn_6_Sn_2_ alloy gradually decreases as the Fe content increases. It is important to note that when the alloy is doped with the fourth element Fe, the Δ*E_1_* value approaches zero, suggesting that the alloy retains good stability even at absolute zero temperatures. The results indicate that the cubic austenite phase in the Ni_7_Fe_1_Mn_6_Sn_2_ alloy is the most stable. Based on this finding, it can also be speculated that when the Fe content exceeds 8 at.%, the transformation from austenite to martensite will not occur in the alloy. On the other hand, the effect of Cu doping on the martensitic transformation is not as pronounced as that of Fe.

Assuming a constant volume of the austenitic and martensitic phases, the ground state of the martensitic phase can be determined by adjusting the tetragonal distortion (c/a ratio). In the calculations, two magnetic structures are taken into consideration, as depicted in Figure 11b–e. In Figure 11b, it is observed that for the Ni_8_Mn_6_Sn_2_ alloy, the total energy of the antiferromagnetic state is significantly lower than that of the ferromagnetic state, suggesting that there exists an antiparallel magnetic interaction between the Sn atomic sites occupied by excess Mn atoms and the original Mn atoms in both the austenite and martensite phases. Using the total energy of the ferromagnetic austenite phase as a reference, it can be observed that as the tetragonal strain increases, the total energy of the alloy in the ferromagnetic state also increases. The curve for the antiferromagnetic state exhibits two energy local minima. The first minimum is found at c/a = 0.9, while the second minimum occurs at c/a = 1.30. Thus, it can be determined that the unmodulated martensite phase, with a c/a ratio of approximately 1.30 represents the most stable phase at very low temperatures.

For the alloys doped with 6.25 at.% and 12.5 at.% Cu, the magnetic states of both austenite and martensite remain unchanged. With the doping of Cu, the c/a value of martensite also increases. In the case of an alloy doped with 6.25 at.% Fe, the total energy of the AFM and FM states almost overlaps at the c/a ratio of 1, suggesting that the ferromagnetism of the austenite phase is enhanced with Fe doping. Additionally, Fe doping reduces the c/a value of the martensite, which is approximately 1.2. The magnetic interaction between Mn-Mn and Mn-Sn still remains in an antiparallel state, indicating that the martensite phase still maintains an antiferromagnetic state. Therefore, a large Δ*M* between the two phases of austenite and martensite could occur.

As listed in Table 1, it can be observed that although the atomic magnetic moment of Cu is small, the total magnetic moment of the martensitic phase in the Ni_7_Cu_1_Mn_6_Sn_2_ alloy shows a slight increase, primarily attributing to the contraction of the unit-cell volume, which reduces the distance between Mn and Ni atoms and enhances their interaction. Consequently, there is an increase in the alloy’s total magnetic moment. The ferromagnetic and antiferromagnetic couplings between Mn-Mn and Mn-Sn are highly sensitive to the distance between them. In the Ni_6_Cu_2_Mn_6_Sn_2_ alloy, the Ni magnetic moment decreases significantly, mainly attributed to the substitution of ferromagnetic Ni atoms with two paramagnetic Cu atoms. As the main contributor to the magnetic moment, the magnetic moment of Mn atoms (approximately 3.48 μB/atom) in the alloy remains relatively unchanged with the doping content of Cu. On the other hand, the magnetic moment of Fe atoms is significantly larger than that of Ni atoms. The Ni_7_Fe_1_Mn_6_Sn_2_ alloy exhibits a noticeable enhancement in total magnetic moment, which is consistent with the analysis presented in Figure 11e, where Fe doping strengthens the ferromagnetism of the alloy.

In summary, the present first-principles calculations have revealed the impact of Cu or Fe doping on *T_M_*, *T_C_*, and the magnetic properties of Ni-Mn-Sn alloys. The results are consistent with those obtained from the DSC and thermomagnetic curves described in Section 3.2, and shed some light on the underlying mechanisms of phase transitions and magnetic properties dependence on Cu- and Fe-doping in the Ni-Mn-Sn-Cu(Fe) alloys. In addition, these calculated results provide an approximate compositional range for the optimization of Ni-Mn-Sn-Cu(Fe) alloys with enhanced magnetic and magnetocaloric properties.

## 4. Conclusions

In conclusion, we systematically investigate the phase transitions and magnetic properties in Ni_50−x_Mn_38_Sn_12_Cu_x_ (x = 0, 2, 3, 4, 5, and 6) and Ni_50−y_Mn_38_Sn_12_Fe_y_ (y = 0, 1, 2, 3, 4, and 5) alloys and establish the structural and magnetic phase diagrams of these alloy systems. As the doping amount of the fourth element increases, the changes in magnetic properties and microstructures of the alloys are elucidated. Martensitic transformation accompanied by changes in magnetization is observed in the alloys with 3 ≤ x ≤ 5 and 2 ≤ y ≤ 4. Doping with appropriate amounts of Cu or Fe enhances the mechanical properties of the alloys and changes their fracture mechanisms. Moreover, the influences of the fourth-element doping on the phase transitions and magnetic properties of the alloys are elucidated by a first-principles calculation. This work demonstrates the significant potential of Cu- or Fe-doped Ni-Mn-Sn-based FSMAs for room-temperature magnetic refrigeration applications, offering remarkable magnetic properties and exceptional mechanical performance. In future studies, more refined doping ratios can be considered, and mechanical and magnetocaloric properties can be further obtained using first-principles calculations, paving a new avenue for designing high-performance multicaloric materials through a combination of experiments and first-principles calculations.

## Figures and Tables

**Table 1:** Lattice constants and total and partial magnetic moments in the cubic austenite and tetragonal martensite phases for Ni_8_Mn_6_Sn_2_, Ni_7_Cu_1_Mn_6_Sn_2_, Ni_6_Cu_2_Mn_6_Sn_2_, and Ni_7_Fe_1_Mn_6_Sn_2_ alloys.

Composition		Lattice Parameters (nm)				Moment (μB)				
		a	c	c/a	Total	Ni	Mn_Mn_	Mn_Sn_	Cu	Fe
Ni_8_Mn_6_Sn_2_	Cub.	5.944797			1.87	0.13	3.48	−3.66		
	Tet.	5.473994	7.011362	1.280849	1.71	0.1	3.37	−3.6		
Ni_7_Cu_1_Mn_6_Sn_2_	Cub.	5.961703			1.79725	0.11	3.481	−3.678	0.003	
	Tet.	5.43446	7.174611	1.320207	1.7795	0.124714	3.38525	−3.566	0.016	
Ni_6_Cu_2_Mn_6_Sn_2_	Cub.	5.978043			1.7175	0.09	3.4785	−3.698	−0.003	
	Tet.	5.396697	7.335354	1.35923	1.6665	0.088333	3.35525	−3.538	0.013	
Ni_7_Fe_1_Mn_6_Sn_2_	Cub.	5.928568			2.1425	0.133429	3.36225	−3.613		1.597
	Tet.	5.588994	6.670863	1.193571	2.0115	0.100857	3.3135	−3.541		1.357

## Data Availability

The original contributions presented in the study are included in the article/Supplementary Materials, further inquiries can be directed to the corresponding authors.

## Supplementary Materials

The following supporting information can be downloaded at: https://www.mdpi.com/article/10.3390/ma17133172/s1, Figure S1: The unit cell of the cubic structure of (**a**) Ni_8_Mn_6_Sn_2_, (**b**) (Ni_7_Cu_1_)Mn_6_Sn_2_, (**c**) (Ni_7_Mn_1_)(Mn_5_Cu_1_)Sn_2_, (**d**) (Ni_7_Sn_1_)Mn_6_(Sn_1_Cu_1_), (**e**) (Ni_6_Cu_2_)Mn_6_Sn_2_, (**f**) (Ni_7_Fe_1_)Mn_6_Sn_2_, (**g**) (Ni_7_Mn_1_)(Mn_5_Fe_1_)Sn_2_ and (**h**) (Ni_7_Sn_1_)Mn_6_(Sn_1_Fe_1_) alloys; Figure S2: Total densities of states for alloys Ni_8_Mn_6_Sn_2_, Ni_7_Cu_1_Mn_6_Sn_2_, Ni_6_Cu_2_Mn_6_Sn_2_, and Ni_7_Fe_1_Mn_6_Sn_2_ with (**a**) austenite and (**b**) martensite phases. The inset is the near-EF total densities of states in the energy range marked by the dot-dashed rectangle. (The Fermi level is indicated by the vertical line at 0 eV).
