# Supplementary material for: Cu- and Fe-Doped Ni-Mn-Sn Shape Memory Alloys with Enhanced Mechanical and Magnetocaloric Properties
Source: Materials (Basel). 2024 Jun 28;17(13):3172. doi: 10.3390/ma17133172 (PMC11242043; doi:10.3390/ma17133172)
Supplement: Supplementary file 1 [file materials-17-03172-s001.zip › materials-3072624-supplementary.pdf]

Supplemental Information

Cu- and Fe-doped Ni-Mn-Sn shape memory alloys with enhanced mechanical and magnetocaloric properties

Siyao Ma[a,b], Xuexi Zhang[a]*, Guangping Zheng[b]*, Mingfang Qian[a], Lin Geng[a]

[a]*School of Materials Science and Engineering, Harbin Institute of Technology, Harbin 150001, China*

[b]*Department of Mechanical Engineering, The Hong Kong Polytechnic University, Hong Kong, China*

*Corresponding authors. E-mail: xxzhang@hit.edu.cn (X. Zhang), mmzheng@polyu.edu.hk (G. Zheng)

(1) The cubic structure of three possible atomic substitutions

In the Cu- or Fe-doped Ni-Mn-Sn alloys, there are three possible atomic substitutions, as follows: (1) Direct substitution of Ni atoms by doping atoms (as shown in Fig. S1(b) and (f)). (2) Doping atoms occupy the positions of Mn atoms, while Mn atoms further replace Ni atoms (as shown in Fig. S1(c) and (g)). (3) Doping atoms occupy the Sn atoms, which in turn replace Ni atoms (as shown in Fig. S1(d) and (h)).

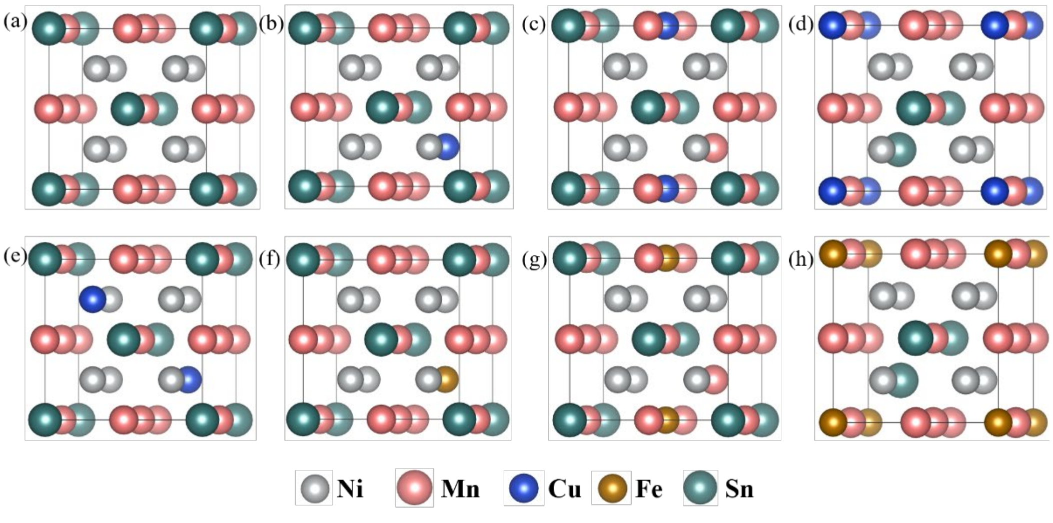

Figure S1. The unit cell of the cubic structure of (a) $Ni_8Mn_6Sn_2$, (b) $(Ni_7Cu_1)Mn_6Sn_2$, (c) $(Ni_7Mn_1)(Mn_5Cu_1)Sn_2$, (d) $(Ni_7Sn_1)Mn_6(Sn_1Cu_1)$, (e) $(Ni_6Cu_2)Mn_6Sn_2$, (f) $(Ni_7Fe_1)Mn_6Sn_2$, (g) $(Ni_7Mn_1)(Mn_5Fe_1)Sn_2$ and (h) $(Ni_7Sn_1)Mn_6(Sn_1Fe_1)$ alloys.

(2) The densities of states for the $Ni_8Mn_6Sn_2$, $Ni_7Cu_1Mn_6Sn_2$, $Ni_6Cu_2Mn_6Sn_2$, and $Ni_7Fe_1Mn_6Sn_2$ alloys

According to the prediction of the alloy's phase transformation temperature, which is based on the difference in total energies of austenite and martensite, the substitution of Ni with the fourth elements Cu or Fe enhances the stability of the alloy's austenite phase, resulting in a decrease in the martensitic transformation temperature. The mechanism is explained by comparing the total density of states (DOS) for Cu- and Fe-substituted Ni. Fig.S2 illustrates the characteristics observed in the total DOS for alloys with various compositions: the spin-up DOS is mainly below E_F, while the spin-down DOS is distributed on both sides of E_F. The distribution of DOS near E_F has a significant impact on the structural stability, bonding characteristics, and martensitic transformation of the Ni-Mn-Sn based alloys.

Upon Cu doping, there is a significant change in the spin-down DOS at E_F. In the undoped $Ni_8Mn_6Sn_2$ alloy, the spin-down DOS is not located at a trough position at E_F, and there is a smaller peak in the spin-down region below E_F. However, when one Cu atom replaces Ni, the peak shifts toward E_F. Upon the substitution of two Cu atoms, the peak disappears, and a trough forms at E_F, creating a pseudogap. These observations indicate that the alloy system becomes increasingly stable with higher Cu content. Similarly, when one Fe atom replaces Ni, there is a trough in the spin-down region at E_F, forming a distinct pseudogap. The results indicate that Fe doping also enhances the stability of the alloy.

Based on the energy band model, the difference in the total number of spin-up and spin-down electrons below E_F determines the change in the total magnetic moment. After doping Fe atoms, there is a slight increase in the number of spin-up electrons below E_F, while the number of spin-down electrons decreases more significantly, which leads to an enhancement of the total magnetic moment in the alloy.

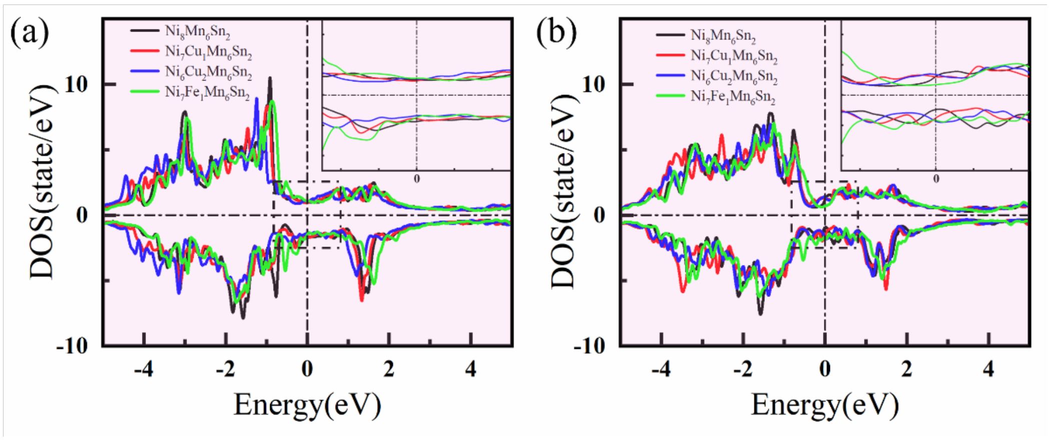

Figure S2. Total densities of states for alloys $Ni_8Mn_6Sn_2$, $Ni_7Cu_1Mn_6Sn_2$, $Ni_6Cu_2Mn_6Sn_2$, and $Ni_7Fe_1Mn_6Sn_2$ with (a) austenite and (b) martensite phases. The inset is the near-E_F total densities of states in the energy range marked by the dot-dashed rectangle.

(The Fermi level is indicated by the vertical line at 0 eV)
